# Myocarditis and COVID-19 related issues

**DOI:** 10.21542/gcsp.2023.28

**Published:** 2023-09-30

**Authors:** Michele Ciabatti, Chiara Zocchi, Iacopo Olivotto, Leonardo Bolognese, Maurizio Pieroni

**Affiliations:** 1 Cardiovascular Department, San Donato Hospital, Arezzo, Italy; 2 Cardiomyopathy Unit, Careggi University Hospital, Florence, Italy; 3 Department of Experimental and Clinical Medicine, University of Florence, Meyer Children Hospital, Florence, Italy

## Abstract

The recent COVID-19 (Coronavirus Disease 2019) pandemic by SARS-CoV2 infection has caused millions of deaths and hospitalizations across the globe. In the early pandemic phases, the infection had been initially considered a primary pulmonary disease. However, increasing evidence has demonstrated a wide range of possible cardiac involvement. Most of systemic and cardiac damage is likely sustained by a complex interplay between inflammatory, immune-related and thrombotic mechanisms. Biventricular failure and myocardial damage with elevation of cardiac biomarkers have been reported in COVID-19 patients, although histological demonstration of acute myocarditis has been rarely documented. Indeed while cardiac magnetic resonance findings include different patterns of myocardial involvement in terms of late gadolinium enhancement, histological data from necropsy and endomyocardial biopsy showed peculiar inflammatory patterns, mostly composed by macrophages. On the other hand COVID-19 vaccines based on mRN technology have been also associated with increased risk of myocarditis. COVID-19 and mRNA vaccine-related myocarditis present different clinical and imaging presentations and recent data suggest the presence of distinctive immunological mechanisms involved.

## Introduction

During the early phases of COVID-19 pandemic, pulmonary involvement was deemed to be the predominant systemic involvement in SARS-CoV-2 infection. Over the course of the pandemic, cardiovascular involvement has emerged as an important negative prognostic factor^1,2^. Increases in troponin, natriuretic peptide and D-dimer levels have been independently associated with increased mortality in COVID-19 patient^1–3^. However, these initial studies were limited by the heterogeneity of the cohorts and by the restricted availability of invasive and advanced evaluation (such as coronary angiography and cardiac magnetic resonance, CMR). Most of these studies focused on moderate and severe COVID-19 patients, often presenting elevated cardiovascular risk profiles and ongoing acute respiratory distress (ARDS) syndrome. These elements reduced the specificity of circulating biomarkers in the definition of cardiovascular involvement in COVID-19 infection.

Myocardial inflammation in patients with COVID-19 has been reported by CMR with evidence of oedema on T2-weighted sequences and presence of both ischaemic and non-ischaemic late gadolinium enhancement (LGE)^4–6^.The advent of COVID-19 vaccines based on messenger ribonucleic acid (mRNA) technology represented a milestone in limiting and mitigating COVID-19 pandemic. However, this class of drugs has been associated with myocarditis onset, especially in young males^7–10^. Clinical and CMR features of mRNA vaccine-related myocarditis differ from COVID-19 myocarditis and present many similarities with classical acute myocarditis^11–14^.

In this review we will discuss the clinical, imaging and immunological aspects of COVID-19 and mRNA vaccine related myocarditis.

## COVID-19-related cardiac damage: Pathophysiological features

**Figure 1.**
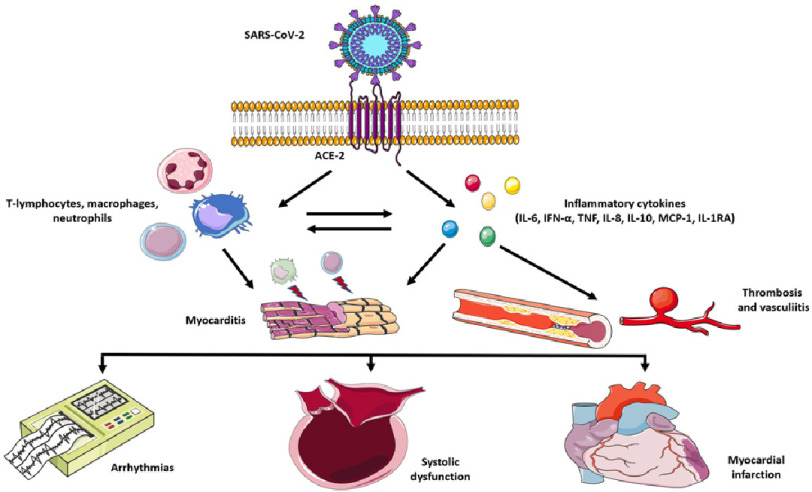
Mechanisms of cardiovascular complications of SARS-CoV2 infection. SARS-CoV-2 enters the cellular membrane due to its affinity to ACE-2 as a receptor. The viral infection determines a marked dysregulated innate immune response with important cellular (T-lymphocytes, macrophages, neutrophils) and cytokine (IL-6, IFN-*α*, TNF, IL-8, IL-10, MCP-1, IL-1RA) activation. These immune and inflammatory processes can provoke myocarditis, thrombosis, and vasculitis in the myocardium. Arrhythmias, biventricular dysfunction and acute coronary syndromes can occur in these patients. **Key:** SARS-CoV-2, severe acute respiratory syndrome coronavirus 2; IL-6, interleukin 6; IFN-*α*, interferon alpha; TNF, tumor necrosis factor; IL-8, interleukin 8; IL-10 interleukin 10; MCP-1, Monocyte chemoattractant protein-1; IL-1RA, interleukin 1 receptor antagonist.

SARS-CoV-2 presents significant vascular tropism due to its binding affinity for angiotensin-converting-enzyme 2 (ACE2) as receptor^15^ (Figure 1).

**Table 1:** Main cardiac histopathological studies in COVID-19 patients.

Study	Population	Design	Pathological findings	Notes
Tavazzi et al., Eur J Heart Fail^33^	1 COVID-19 patients with ARDS and cardiogenic shock undergoing circulatory mechanical support	The patient underwent EMB. Histological and ultrastructural analysis were performed.	Evidence of interstitial and endocardial inflammatory infiltrates (CD68+ macrophages). Presence of viral particles in interstitial cells with signs of cytopathic damage.	Presence of mild interstitial and perivascular fibrosis.
Bradley et al., Lancet^14^	14 patients deceased due to severe COVID-19	All patients underwent lung, cardiac and kidney autopsies. Histological, Immunohistochemistry and ultrastructural evaluations were performed.	Presence of cardiac fibrosis in 100% of subjects and myocyte hypertrophy in 93% of them. Evidence of lymphocytic myocarditis in 1 patient with myocyte necrosis.	Immunohistochemical analysis for SARS-CoV-2 in cardiac samples turned out to be negative.
Wichmann et al., Ann Intern Med^15^	12 consecutive subjects deceased due to severe COVID-19	Autoptic cardiac and pulmonary evaluation.	Evidence of RV lymphocytic myocarditis in 1 patient.	Massive pulmonary thrombosis in 4 cases, presence of DVT in 3 subjects.
Lax et al., Ann Intern Med^16^	48 patients deceased due to severe COVID-19	All subjects underwent lung, heart, liver and kidney autopsies.	Presence of enlarged myocytes with nuclear polymorphism. 10/48 subjects presented patchy fibrosis. Presence of lymphocytic infiltrates in 1 case.	Lung autopsies demonstrated presence of extensive parenchymal and intravascular inflammatory infiltrates with frequent signs of thrombosis.
Buja et al., Cardiovasc Pathol^17^	23 deceased patients due to severe COVID-19	Cardiac, pulmonary, and splenic autopsies of the included subjects.	Viral localization in the cardiac interstitial and perivascular cells. Evidence of small vessel vasculitis. Lymphocytic myocarditis in 1 case.	Presence of ARDS and mononuclear infiltrates in lung autopsies. Evidence of large and small pulmonary vessel thrombosis.
Escher et al., ESC Heart Fail^34^	104 patients undergoing EMB due to suspected myocarditis or unexplained heart failure during early pandemic phases.	All patients underwent EMB with immunohistological evaluation and qRT-PCR for SARS-CoV-2 genome.	Positive qRT-PCR ofr SARS-CoV-2 in 5/104 EMBs. Presence of active myocarditis following Dallas criteria in 1/5 patients. Presence of elevated levels of T-cless, macrophages, lymphocytes and memory T-memory cells in 4 subjects.	All COVID-19 patients presented increased levels of cell adhesion molecules. Presence of vasculitis in one patient.
Weckbach et al., Circ Cardiovasc Imaging^48^	18 subjects with SARS-CoV-2 infection and increased cardiac damage biomarkers.	All patients underwent CMR. EMB performed in 5/18 patients.	5/5 patients presented lymphocytic myocarditis at histology with no evidence of SARS-CoV-2 genome on qRT-PCR.	CMR demonstrated active myocarditis in only 38.9% of subjects following revised Lake-Louise criteria.
Tanacli et al., Front Cardiovasc Med^13^	32 patients with persisting cardiac symptoms after SARS-CoV-2 infection.	All subjects underwent CMR. 10/32 patients underwent EMB.	Presence of fibrosis and mild macrophage infiltration in 5/10 subjects.	Presence of active myocarditis in only 9% of COVID-19 patients following revised Lake-Louise criteria at CMR.
Weckbach et al., JAMA Cardiol^35^	5 patients with SARS-CoV-2 infection.	All patients underwent EMB, immunohistochemical, immunofluorescence proteomic and RNA analysis.	Predominant macrophage infiltration in COVID-19 EMBs and significant activation of serine/threonine kinase, MAPK and complement pathways.	Increased macrophage co-expression of the CD-163 scavenger receptor (also expressed by pulmonary macrophages in COVID-19 pneumonia).

**Notes.**

COVID-19 coronavirus disease 2019 ARDS acute respiratory distress syndrome EMB endomyocardial biopsy SARS-CoV-2 severe acute respiratory syndrome coronavirus 2 RV right ventricle DVT deep venous thrombosis q-RT-PCR quantitative real time polymerase chain reaction CMR cardiac magnetic resonance RNA ribonucleic acid MAPK mitogen activated protein kinases

**Figure 2.**
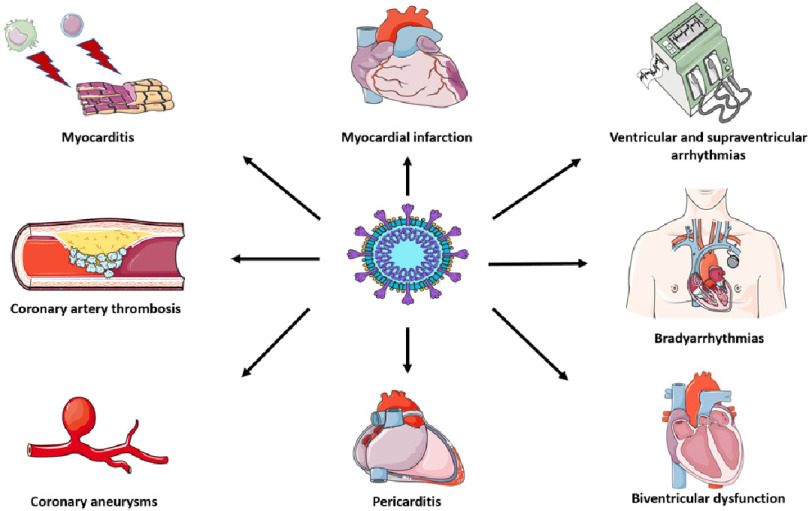
Cardiovascular manifestations of SARS-CoV2 infection. SARS-CoV-2 mainly causes macrophage and lymphocytic myocarditis, while its direct pathogenetic role is still debated. Due to its peculiar vascular tropism, many cases of arterial and venous thrombosis and coronary vasculitis have been described in literature. These pathogenetic mechanisms can lead to biventricular dysfunction, brady and tachyarrhythmias and myocardial infarction (even with non-obstructive coronary arteries). Pericarditis is another possible complication of COVID-19 infection. **Key:** COVID-19, coronavirus disease 2019; SARS-CoV-2, severe acute respiratory syndrome coronavirus 2.

**Figure 3.**
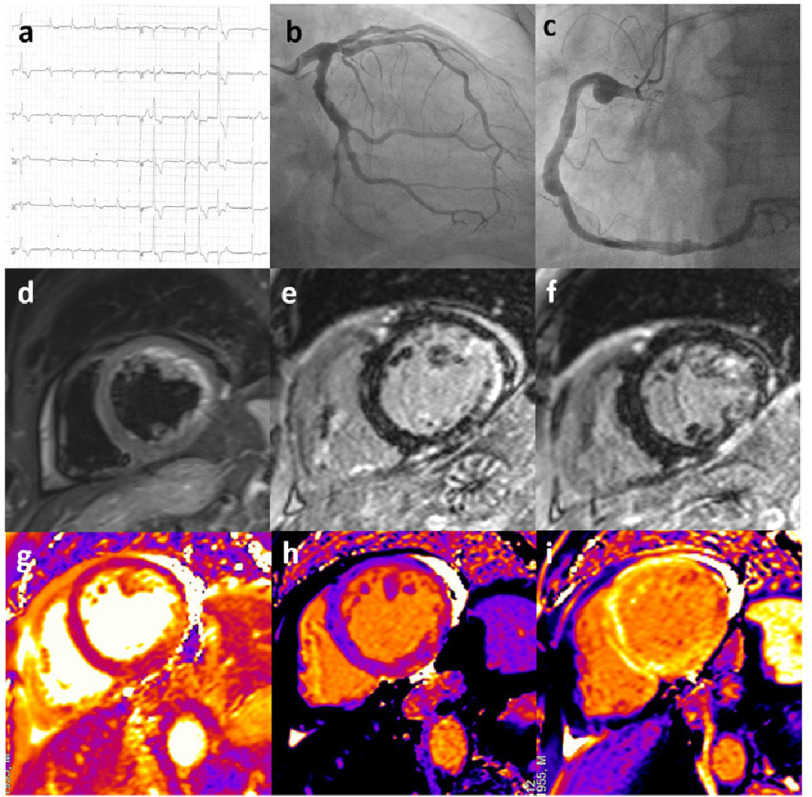
A case of COVID-19-related cardiovascular damage. A 65-year-old male presenting with severe left ventricular dysfunction and non-sustained ventricular tachycardia shortly after hospitalization due to COVID-19 pneumonia. (a) ECG at presentation showed the presence of sinus rhythm with normal atrioventricular conduction, left anterior hemiblock, QRS fragmentation in V1–V2 leads and negative lateral T waves; polymorphic ventricular beats were present. (b–c) Coronary angiography showed diffuse aneurysmatic lesions in the absence of critical stenosis in the circumflex (b) and right (c) coronary artery. (d) CMR demonstrated the presence of myocardial oedema in the LV inferolateral walls on T2-weighted images (short-tau inversion recovery, STIR) with mild pericardial effusion. (e–f) Late sequences after contrast administration showed the presence of extensive ischaemic LGE in the mid LV lateral wall and midwall LGE in the apical anterolateral wall. LGE was detected also in the anterior RV wall. (g) T2 mapping confirmed the presence of inflammation in the lateral LV wall. h and i: Image analysis of Native and post-contrast T1 mapping (ShMOLLI at 1.5 T) showed increased T1 and ECV values matching LGE sequences in the inferior and lateral wall. **Key:** COVID-19, coronavirus disease 2019; CMR, cardiac magnetic resonance; LV, left ventricle; STIR, short-tau inversion recovery; LGE, late gadolinium enhancement; RV, right ventricle; ECV, extracellular volume.

Lung autopsies performed in the early pandemic phases showed presence of viral localization in the pulmonary vessels with consequential endothelial damage and intussusceptive angiogenesis^16^. Multiple studies demonstrated marked dysregulation of the innate immunity leading to significant cytokine (such as IFN-*α*, TNF, IL-6, IL-8, IL-10, MCP-1, IL-1RA) release, complement and neutrophil activation^17–20^.

Cytokine storm syndrome has emerged as a hallmark feature of moderate and severe COVID-19 infections, leading to multiorgan failure and worse outcome^18,21,22^. Accordingly, multisystemic inflammatory syndrome in children (MIS-C) represents a serious complication of SARS-CoV-2 infection in the paediatric population^23^.

Left ventricular dysfunction, haemodynamic instability, troponin release, pericardial effusion and coronary aneurysms have been reported in MIS-C^24^. Of note these aneurysms may completely regress after treatment with steroids^25^. Rare reports of coronary aneurysms possibly related to concomitant or recent SARS-CoV2 infection have been described also in adults^26^.

Taken together, these data suggest that most of COVID-19 related damage could be mediated by direct and indirect effects of marked dysregulated immune response rather than direct viral damage. Evidence of direct myocardial damage by SARS-CoV2 is limited to some cases demonstrating viral localization in interstitial cells and positive genome analysis in endomyocardial biopsies (EMBs)^27,28^.

Beta-coronaviruses, including SARS-CoV-2, use ACE-2 as cell entry by its receptor-binding domain (RBD)^15^ and this element could explain its preferential vascular tropism. Bradley et al. described the presence of myocardial fibrosis in 14 patients undergoing autopsies after severe COVID-19 infection with evidence of lymphocytic infiltrates in only one case^29^. Notably, immunohistochemistry did not detect SARS-CoV-2 in any tissue sample. Other authors reported the presence of extensive inflammatory and thrombotic processes in 12 COVID-19 lung autopsies, but they detected right ventricular (RV) lymphocytic myocarditis in only one case^30^.

Similarly, Lax et al. described one case of lymphocytic infiltrates in 48 cardiac autopsies with evidence of fibrosis in 10 patients^31^. Another group reported only one case of lymphocytic myocarditis in 23 COVID cardiac autopsies^32^ presenting viral particles in interstitial and perivascular cells. Moreover, small vessel vasculitis was found in some tissue samples. The presence of SARS-CoV-2 viral genome was detected by quantitative polymerase chain reaction in 5/104 subjects undergoing EMBs in the early pandemic phase^28^. Infiltrates of lymphocytes, macrophages and memory T cells were detected, but the Dallas criteria for active myocarditis were reached in only one case. Tanacli et al. reported the presence of fibrosis and macrophage infiltration in 5/10 subjects undergoing EMB^5^. Lymphocytic infiltrates were found in 5/5 COVID-19 patients undergoing EMB due to the presence of increased cardiac biomarkers by Weckbach et al.^33^. Notably, CMR was able to detect active myocarditis in only 39% of subjects. Moreover, the same group described the presence of predominant T-lymphocytes and macrophages infiltrates and prominent complement and mitogen activated protein kinase (MAPK) pathways activation in COVID-19 EMBs^34^ (Table 1).

## COVID-19 CARDIAC DAMAGE: CLINICAL, IMAGING AND PATHOLOGICAL FINDINGS

### Clinical presentation and imaging findings

Left and right ventricular dysfunction are relatively common in severe COVID-19 and have been associated with worse outcomes in literature^35–38^. Ammirati et al. reported high rates (39%) of fulminant presentation in a cohort of COVID-19 myocarditis^39^. Presence of concomitant pneumonia has been associated with worse prognosis in patients with COVID-19 myocarditis^39^. Ventricular arrhythmia, high-grade atrioventricular block, pericardial effusion and cardiac tamponade can complicate the clinical course^26^. Notably, Barhoum et al. reported lower left ventricular ejection fraction, increased use of mechanical circulatory support and higher intensive care unit complications in COVID-19 myocarditis without multisystem inflammatory syndrome (MIS-) compared to MIS+ subjects (Figure 2)^25,39^.

Electrocardiographic signs of RV strain, presence of left bundle branch block, QRS fragmentation, repolarization abnormalities and supraventricular arrhythmias have been associated with a worse prognosis in COVID-19 patients^40–43^. Advanced atrioventricular block and ventricular arrhythmias are relatively uncommon and limited to most severe cases^26^.

Echocardiography can demonstrate the presence of right and left ventricular dysfunction, reduced biventricular strain and pericardial effusion^26,35–39^. CMR studies performed in patients hospitalized for COVID-19 demonstrated the presence of ischaemic LGE patterns in 6–22% of patients, even in patients without significant lesions at coronary angiography^4,44^. Coronary vasculitis and biventricular dysfunction have been described in paediatric patients with COVID-19 related MIS-c^23,24^. Presence of multisystemic inflammatory syndrome has been reported also in the adult population (MIS-A)^26^.

Presence of oedema and non-ischaemic LGE patterns compatible with myocarditis have been reported in a significant proportion of patients after SARS-CoV-2 infection^4,5,44,45^. Although the authors could not exclude the presence of concomitant inherited or acquired cardiac diseases in all cases, the relatively high prevalence of these alterations and the frequent association with CMR signs of oedema (in terms of positive T2-weighted or T2 mapping sequences) suggest a pathogenetic role of SARS-Cov-2 infection. Ammirati etal.reported high rates of biventricular dysfunction in patients with COVID-19 related myocarditis with frequent need of vasoactive drugs and/or mechanical support^39^. A case of severe COVID-19 patient with complex cardiovascular involvement is described in Figure 3.

Cardiac screening performed on athletes recovering from SARS-CoV-2 infection revealed the presence of cardiac abnormalities in 0.7−2.3% of subjects by use of CMR in terms of pathological T2-derived sequences, presence of increased native T1 mapping and non-ischaemic LGE^46,47^. Patients with cardiopulmonary symptoms and abnormal ECG, stress testing or echocardiographic findings more likely presented pathological findings at CMR.

### Histology and proposed mechanisms

Cardiac autopsies in COVID-19 patients demonstrated the presence of fibrosis and macrophage infiltration in tissue samples with low prevalence of active lymphocytic myocarditis following Dallas criteria^29–32,48^. Small vessel vasculitis has been detected in a cardiac autoptic COVID-19 cohort^32^.

EMBs demonstrated the presence of SARS-CoV-2 viral genome in only a minority of cases^27,28,39^. Macrophages, neutrophils and T lymphocytes were the main cellular lines found in tissue samples and many cases did not reach the classical Dallas criteria for the histological diagnosis of acute myocarditis^33,34,39^. The low prevalence of pathological infiltrates could be potentially explained by sample biases related to EMB technique, especially when performed only in the RV^49^. However, these findings were concordant across multiple studies, suggesting the presence of peculiar pathological features in COVID-19 related myocarditis. These elements, together with the evidence of MAPK and complement pathways activation, further support the hypothesis of a predominant immune response activation in patients with COVID-19 cardiac involvement.

### Management and outcome

Treatment of hospitalized patients with SARS-CoV-2 infection is based on corticosteroids, heparin and remdesivir^50–52^. The important role of cytokines in the pathogenesis of COVID-19 related systemic damage led many groups to use specific immunosuppressive drugs in the management of these patients. Use of interleukin-6 (IL-6) inhibitors has been associated with mixed results in literature^53^ although a recent meta-analysis reported improved outcomes in COVID-19 patients receiving IL-6 inhibitors in combination with steroid therapy^54^. Interestingly, the use of baricitinib (a janus kinase inhibitor) in combination with remdesivir has been associated with improved outcomes compared to remdesivir alone in a randomized controlled trial^55^. Steroid therapy is recommended in case of COVID-19 related myocarditis with heart failure, MIS-A or haemodynamic instability^56^. Ammirati etal. reported the use of remdesivir, IL-6 inhibitors and intravenous immunoglobulins in a large cohort of patients with COVID-19 related myocarditis^39^. Anecdotal use of immunoadsorption in severe cardiac and pulmonary COVID-19 infection has been also described^57^.

## COVID-19 VACCINES AND MYOCARDITIS

COVID-19 vaccines based on messenger RNA technology have played a crucial role in limiting and mitigating the pandemic due to their efficacy and safety profile^58–61^. The registration trials did not demonstrate serious adverse effects, but many cases of myocarditis have emerged after widespread vaccination^10–13^. COVID-19 vaccines-related myocarditis occurred in young males, usually a few days after second dose administration^10–13,62^. Estimated prevalence ranges from 0.2 to 38.9 cases per million doses, depending on age, sex and geographical distribution^12,13,63^.

### Clinical presentation and imaging findings

Chest pain is the most typical symptom, sometimes associated with fever and flu-like symptoms^62^. Heart failure and cardiogenic shock are uncommon (less than 10% of cases)^12^. Ammirati etal.reported the presence of ST segment elevation in 60% of cases and non-sustained ventricular tachycardia in 11% of subjects^62^. Most of the patients presented preserved biventricular function with pericardial effusion in 16% of them. CMR demonstrated the presence of oedema on T2-weighted images and T2 mapping sequences^14,62,64^. Signs of oedema, late gadolinium enhancement and increased T1 mapping values were mostly observed in inferolateral and anterolateral segments^14,62,64^.

### Histology and proposed mechanisms

EMBs performed in patients with clinically suspected vaccine-related myocarditis demonstrated the presence of B and T lymphocytes, plasma cells, macrophages and degranulated eosinophils^65–67^. Notably, EMBs revealed the presence of chronic healed myocarditis and sarcoidosis in patients with clinically suspected COVID-19 vaccine related myocarditis^66^.

**Figure 4.**
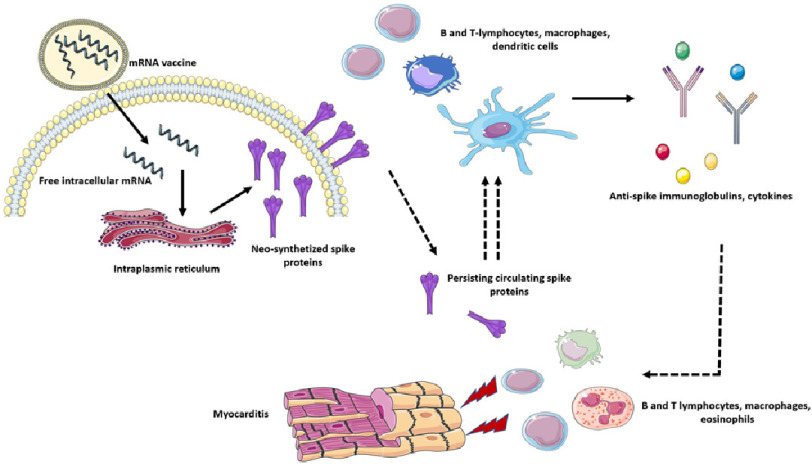
Proposed mechanisms of COVID-19 mRNA vaccine-related myocarditis. COVID-19 vaccines based on mRNA technology enter cells by endocytosis and release their mRNA (coding for COVID-19 spike protein) content in the cytoplasm. The mRNA is therefore transcripted in the endoplasmic reticulum into numerous spike proteins. These molecules are then exposed in the outer cellular membrane and presented to B and T-lymphocytes, dendritic cells, dedicated macrophages and other antigen presenting cells. The antigen exposure and recognition lead to marked immune cells activation and expansion with consequent immunoglobulin and cytokine production. It has been proposed that persistent free circulating spike proteins could determine an excessive and dysregulated adaptive immune response. These immune pathways could therefore play a role in determining myocarditis with extensive lymphocytes, macrophages and eosinophilic infiltrates. **Key:** COVID-19, coronavirus disease 2019; mRNA, messenger ribonucleic acid.

The mechanisms leading to myocarditis following mRNA vaccine administration remain largely unclarified. An exaggerated immune response to vaccine has been claimed but not conclusively demonstrated^65^. Interestingly, a recent work by Yonker and colleagues^68^ demonstrated that patients with myocarditis following mRNA vaccine administration present high levels of unbound circulating spike protein compared to vaccinated controls without myopcarditis. Extensive immunophenotype characterization also demonstrated the presence of increased effector memory T cells and PD-1–expressing bulk CD4+ T cells in patients with myocarditis, suggesting a potential exhaustion in these cellular lines. Moreover, the author reported increased levels of IL-8, IL-6, tumor necrosis factor-*α*, IL-10, interferon-*γ*, and IL-1 *β* and lower IL-4 levels in subjects with myocarditis compared to controls (Figure 4).

Compared to COVID-19 myocarditis, vaccine-related myocarditis present a milder clinical course with frequent infarct-like presentation (Table 2). CMR usually demonstrates preserved biventricular function, while COVID-19 myocarditis can be frequently associated with RV or LV impairment. Oedema and non-ischaemic LGE has been usually detected in the inferolateral segments, similarly to classical viral myocarditis. CMR signs of ischaemic scars have not been described in these patients, differently from COVID-19 myocarditis. Tissue samples obtained from EMB demonstrated florid lymphocytic, macrophage and eosinophilic infiltrates, while COVID-19 EMBs usually present scarce macrophage infiltration and rare cases of lymphocytic myocarditis (Table 2).

**Table 2:** Comparison between COVID-19 related and COVID-19 mRNA vaccine-related myocarditis: clinical, imaging and pathological features.

COVID-19 related myocarditis	COVID-19 mRNA vaccine-related myocarditis
**Pathophysiology**	
• Innate immunity dysregulation and cytokine pathways activation • Direct viral damage (limited evidence)	• Persistent elevated circulatory levels of spike protein (limited data) • Increased expression of inflammatory cytokines and activated T lymphocytes (limited data)
**Clinical presentation**	
• Acute myocarditis (infarct-like, arrhythmic, heart failure or fulminant presentation) • Chronic inflammatory cardiomyopathies and long-term biventricular dysfunction	• Infarct-like presentation (most common) • Heart failure/fulminant myocarditis (uncommon) • Serious brady-tachyarrhythmia (uncommon)
**ECG**	
• Signs of RV strain • Bundle branch blocks • Fragmented QRS • Repolarization abnormalities • Atrial fibrillation/supraventricular arrhythmia • Ventricular arrhythmia • Bradyarrhythmia	• ST segment alterations • NSVTs • Sustained ventricular arrhythmia (uncommon)
**Imaging**	
• **Echocardiography**: RV and LV dysfunction, reduced biventricular strain, coronary aneurysms in MIS-A/MIS-C • **CMR**: biventricular dysfunction, signs of oedema on T2-weighted sequences, ischaemic and non-ischaemic LGE	• **Echocardiography**: LV/RV wall motion abnormalities, LV/RV dysfunction (uncommon) • **CMR**: LV/RV dysfunction (uncommon), signs of oedema on T2-weighted sequences, non-ischaemic LGE
**Histology**	
• SARS-Cov-2 localization in the cardiac interstitial space (limited data) • Limited amount of oedema in tissue samples from autopsies or EMBs • Limited T-lymphocyte and macrophages infiltrates • MAPK and complement pathways activation	• Infiltrates of B and T lymphocytes, plasma cells, macrophages and degranulating eosinophils
**Treatment**	
• Inotropes and mechanical support in case of haemodynamic instability • Corticosteroids • Antivirals (remdesivir) • Heparin (in case of moderate-to-severe systemic COVID-19) • IL-6 inhibitors (contrasting evidence) • Janus kinase inhibitors (limited evidence) • Immunoglobulins (limited data) • Immunoadsorption (anecdotal)	• Inotropes and mechanical support in case of haemodynamic instability (uncommon) • Corticosteroids and immunosuppressive therapies (limited data)

**Notes.**

COVID-19 coronavirus disease 2019 mRNA messenger ribonucleic acid RV right ventricle NSVT non sustained ventricular tachycardia MIS-A multisystem inflammatory syndrome in adults MIS-C multisystem inflammatory syndrome in children CMR cardiac magnetic resonance LGE late gadolinium enhancement LV left ventricle SARS-CoV-2 severe acute respiratory syndrome coronavirus 2 EMB endomyocardial biopsy MAPK mitogen activated protein kinase IL-6 interleukin-6

### Management and outcome

**Figure 5.**
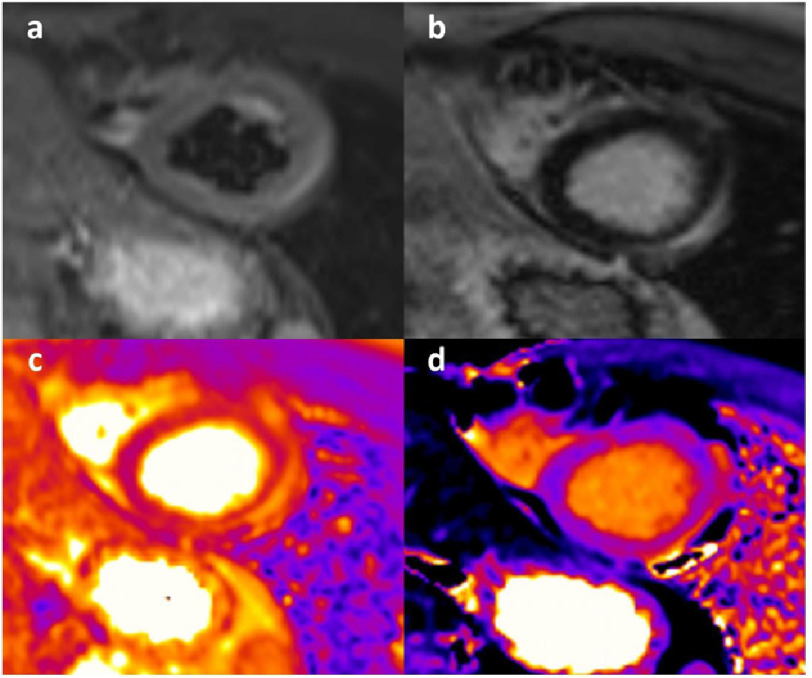
A case of CIVID-19 mRNA vaccine-related acute myocarditis. A 23-year-old male presenting with chest pain and elevation of markers of cardiac damage 3 days after the administration of a second dose of mRNA vaccine. (a) STIR sequences showed presence of subepicardial oedema in the mid-apical wall. (b) late sequences after contrast demonstrated corresponding LGE in the same segments. (c) T2 mapping sequences confirmed the presence of inflammation in the lateral wall. (d) presence of increased T1 mapping values in the corresponding segments on native T1 mapping sequences. **Key:** mRNA, messenger ribonucleic acid; STIR, short-tau inversion recovery; LGE, late gadolinium enhancement.

From a clinical point of view, most of these patients present a benign course with low rates of fulminant myocarditis or sustained ventricular arrhythmias^10–13,62^. Ammirati et al. reported persistence of oedema in only 20% of subjects and significant LGE reduction at 3-months CMR follow-up, in line with imaging findings of classical myocarditis^62^. A case of acute myocarditis following mRNA vaccination is illustrated in Figure 5.

Rare cases of fulminant myocarditis with haemodynamic instability should be treated with inotropes and mechanical support^24,66^. The use of corticosteroids, colchicine and nonsteroidal anti-inflammatory drugs has been described in literature^62,69^, but comparative efficacy data are still lacking.

It has been debated whether patients with a previous non-COVID-19-related myocarditis could present a higher risk of myocarditis recurrence following COVID-19 vaccination. Pieroni et al. reported no cases of COVID-19 vaccine related myocarditis in a cohort of patients with a history of a previous acute myocarditis undergoing mRNA COVID-19 vaccination^70^. These preliminary data suggest that a clinical history of previous acute myocarditis does not portend per se an increased risk for myocarditis following mRNA-based vaccination.

## CONCLUSIONS

Myocardial damage in COVID-19 can be extremely heterogeneous and only in a subset of cases can be properly classified as myocarditis. While CMR can frequently show signs of acute and chronic myocarditis in patients with COVID-19, in these subjects histologic findings usually differ from those obderved in classical myocarditis, while a direct SARS-CoV-2 myocaridal damage is still debated.

COVID-19 vaccines based on mRNA technology have been associated with an increased risk of myocarditis, especially in young males. In these patients CMR findings are comparable to those observed in classical myocarditis, while histology demonstrated the presence of lymphocytic and eosinophilic infiltrates. Most patients present mild forms with a favourable outcome also in terms of residual scar at follow-up CMR, with a possible beneficial effect of steroids and immunomodulatory drugs being reported.
